# Supplementary figures and images for: Categorical consistency facilitates implicit learning of color-number associations
Source: PLoS One. 2023 Jul 10;18(7):e0288224. doi: 10.1371/journal.pone.0288224 (PMC10332609; doi:10.1371/journal.pone.0288224)

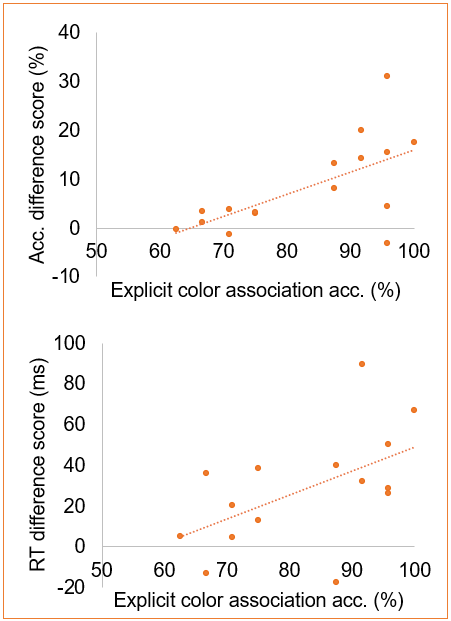

Supplement: S1 Fig — In the category-level experiment, individuals’ explicit color association task accuracy (acc.) was significantly correlated with their difference scores in the main experiment, for both accuracy (congruent–incongruent) and RT (RT: incongruent–congruent). (TIF) [file pone.0288224.s001.tif]
